# Associations of non-employment with common mental disorder subcomponents among working age population: Analysis of national data from 1993, 2000, 2007 and 2014

**DOI:** 10.1177/00207640241293351

**Published:** 2024-11-14

**Authors:** Gayan Perera, KTDDP Jayapala, Mizanur Khondoker, Karen Glaser, Giorgio Di Gessa, Robert Stewart

**Affiliations:** 1 Department of Psychological Medicine, Institute of Psychiatry, Psychology and Neuroscience, King’s College London, UK; 2 South London & Maudsley NHS Foundation Trust, UK; 3 Faculty of Medicine, University of Moratuwa, Sri Lanka; 4 Norwich Medical School, University of East Anglia, Norwich, UK; 5 Institute of Gerontology, Department of Global Health & Social Medicine, King’s College London, UK; 6 Department of Epidemiology & Public Health, Institute of Epidemiology & Health, University College London, UK

**Keywords:** Employment status, British National Surveys of Psychiatric Morbidity, common mental disorder, panic symptoms

## Abstract

**Background:**

Associations between employment status and mental health are well-recognised and such associations may have multiple modifying factors which may also contribute to variations in results.

**Aims:**

We aimed to investigate associations between non-employment and CMD subcomponents and the extent of their variation across age groups using nationally representative data in Britain.

**Method:**

We used a series of national mental health surveys of adults living in private households: the British National Surveys of Psychiatric Morbidity of 1993, 2000, 2007 and 2014. Employment status was the primary exposure of interest. Presence or absence of each fourteen symptoms of common mental disorder (CMD), as the primary outcome, was ascertained identically in all surveys from the revised Clinical Interview Schedule (CIS-R). Odds ratio for the association between exposure and outcome and population attributional fractions (PAFs) for each association was calculated.

**Results:**

Within the highest-risk 45 to 54 years age group, all odds ratios were statistically significant and strongest associations were observed with panic symptoms (OR = 2.33), followed by depressive symptoms (1.90), worry about physical health (1.84), depression (1.82), forgetfulness (1.82) and somatic symptoms (1.70). In the 55 to 64 years age group, highest population attributable fractions were observed for non-employment as a hypothetical risk factor for panic symptoms (51.7%), phobias (44.2%), forgetfulness (39.5%), depressive symptoms (38.5%), worries about physical health (37.9%) and somatic symptoms (36.0%).

**Conclusions:**

The particularly high impact in middle-aged, pre-retirement groups of non-employment on CMD suggests a policy focus on alleviating stressors and providing support for those made redundant and/or compelled to take unwanted early retirement.

## Introduction

One in six people aged above 16 years living in England has symptoms of a mental disorder (Baker, 2020) with 75% not receiving proper treatment (Davies, 2014) and wait times for psychological therapy varying between 4 to 61 days (Baker, 2020). Associations of non-employment with poor mental health are well-recognised, with many studies reporting a higher prevalence of mental disorders in non-employed groups, including depression (Bedaso et al., 2021; Picchio et al., 2019), sleep-related problems (Palmer et al., 2017; Wolińska, 2020), cognitive symptoms (Xue et al., 2017) and generally with common mental disorders (CMD; Achdut et al., 2020; Batic-Mujanovic et al., 2017; Cygan-Rehm et al., 2017; Frasquilho et al., 2016; Honkonen et al., 2007; Park et al., 2016; Paul et al., 2009; Picchio et al., 2019). A few studies have reported a positive association of non-employment with mental health under some circumstances (Eibich, 2015; Gory et al., 2018; Vahtera et al., 2009), including a reduction of antidepressant use following retirement (Oksanen et al., 2011). Different CMD components evaluated by different studies may underlie this heterogeneity in findings, but few studies have focussed on individual symptoms, and these on a limited number. For example, being unemployed or experiencing health-related non-employment is associated with higher suicide risk, compared to the employed (Øien-Ødegaard et al., 2023). The odds of distress is 1.8 to 3.1 times higher in the non-employed than in the employed (Honkonen et al., 2007). Individuals with a sleep disorder were significantly more likely to be non-employed (Huyett & Bhattacharyya, 2022). Anxiety disorders and depressive episodes were high among non-employed compared with employed (Dobson et al., 2020).

Associations between non-employment and CMD risk may have multiple modifying factors which may also contribute to variations in results. For example, non-employment has been found to have a higher impact in men than women (Buffel et al., 2015; Caetano et al., 2016; Ford et al., 2010; Starace et al., 2017; Strandh et al., 2013; Picchio et al., 2019), in blue-collar compared to white-collar jobs (Paul et al., 2009); its impact may also be lessened or even positive in physically demanding (Mazzonna et al., 2017) and poor quality jobs (Butterworth et al., 2012), but may be increased in countries with lower economic development, uneven income distributions and/or poor unemployment protection setup (Paul et al., 2009), as well as following economic crisis (Bartoll et al., 2014; Hauksdóttir et al., 2013). Furthermore, the cause of non-employment is important: stronger associations with poor mental health have been found when work has been stopped on health grounds (Artazcoz et al., 2010; Vahtera et al., 2009) or due to redundancy (Batic-Mujanovic et al., 2017; Dave et al., 2006). Associations between non-employment and CMD might be expected to vary across age groups, although these modifiers have received little investigation.

Therefore, our study aims to investigate associations between non-employment and CMD subcomponents, and how this relationship varies across age groups, taking advantage of pooled data in a series of nationally representative surveys with a robust, detailed and widely used measure of CMD carried out in England between 1993 to 2014.

## Methods

### Data and sample

The data for these analyses were drawn from a series of national mental health surveys of adults living in private households: the British National Surveys of Psychiatric Morbidity of 1993, 2000, 2007 and 2014. The most recent 2007 and 2014 surveys were conducted by the National Centre for Social Research (NatCen) in collaboration with the University of Leicester while earlier surveys were carried out by the Office for National Statistics. The 1993, 2000 survey was carried out in England, Wales and Scotland, whereas the 2007 and 2014 surveys were based in England only. The lower age limit for participation was 16 years for all four surveys, but the upper age limit varied: 64 for the 1993 survey, 74 for the 2000 survey and no upper limit for the 2007 and 2014 surveys. The surveys have sought to retain identical core measures to maximise the comparability of results.

The sampling methodology has been comparable across surveys, employing independent random sampling across the geographic areas in question, not recruiting previous participants or sampling from identical areas. In each survey, primary sampling units (postal sectors) were selected from the Small Users Postcode Address File, stratified for region and social class composition to generate a nationally representative sample. Households were randomly selected from within each sampling unit, and in households containing at least one member in the age range for that survey, one person was randomly selected and invited to participate. Each person was only interviewed once. In 1993, 12,730 participants were interviewed (79.4% participation); In 2000, 8,580 participants were interviewed (67.1%); in 2007, 7,403 participants were interviewed (56.2%); and in 2014, 7,508 participants were interviewed (57.2%). Analyses described here were restricted to residents in England, to maximise comparability across the four waves. The total analysed sample contained 28,248 participants from the four surveys combined (8,565 in 1993, 7,025 in 2000, 7,090 in 2007 and 5,568 in 2014).

### Variables

Employment status was the primary exposure of interest. In each survey, participants were asked whether they had carried out any paid work in the preceding week, with responses to further questions on income earned from employment or self-employment used to cross-check employment status categorisation.

Common mental disorder (CMD), as the primary outcome, was ascertained identically in all surveys from the revised Clinical Interview Schedule (CIS-R; Lewis et al., 1992), which is a widely used, fully-structured questionnaire with stem and supplementary questions enquiring in detail about the following 14 symptoms in the past week: somatic, fatigue, concentration/ forgetfulness, sleep problems, irritability, health worry, depression, depressive symptoms, general worry, anxiety, phobias, panic, compulsions and obsessions. Each symptom schedule generates a 0 to 4 or 0 to 5 score based on frequency, duration and severity in the preceding week.

Based on previous research findings we extracted covariates which were recognised as being associated with either or both paid work and CMD, and which had been ascertained in an identical manner in both surveys. These included the following socio-demographic covariates: age, gender, marital status, education, occupational social class, housing tenure, self-reported financial difficulty, smoking status and physical health. Marital status was grouped into three categories: divorced/separated/widowed, married/cohabiting, single and widowed. Respondents’ highest educational qualifications were grouped into three categories: 9i) A-level and above [implying a school leaving age of 18 years], (ii) GCSE/GCE/O-level [implying a school leaving age of 16 years] and (iii) no qualifications. Social class was determined by the respondents’ primary occupation or most recent occupation (categorised into Professional/ managerial/ clerical; Skilled/ partly-skilled occupations; and Unskilled occupations); those categorised as ‘Armed forces’ (193 respondents) or ‘Never worked’ (7,182 respondents) were excluded). Housing tenure was categorised into four groups: (i) owned with a mortgage, (ii) owned outright, (iii) privately rented and (iv) social housing. In addition, serious financial difficulty was defined as either a reported financial crisis in the previous 6 months or being behind with any payments. Self-reported smoking status was grouped into never smoked, ex-smoker and current smoker categories. Self-reported physical conditions had been ascertained using different approaches in both surveys and these measures were considered too heterogeneous to use in this analysis. However, in all surveys participants were asked whether they had consulted a general practitioner in the previous 12 months for a physical health problem which was coded as a binary variable, and this was supplemented by scores for an activities of daily living (ADL) scale, identically administered in all both surveys and enquiring about difficulties in the following domains on a 0 to 2 scale: (i) personal care; (ii) using transport; (iii) medical care; (iv) household activities; (v) practical activities; (vi) paper work and (vii) money. Because of the distribution of scores in the sample age group, a binary variable was created indicating some difficulty or lot of difficulty for at least one ADL domain.

### Statistical analysis

Consistent with previous cross-survey publications, analyses were not weighted, as surveys used different weighting precluding comparisons of estimates over time. Having described distributions of CMD components by sociodemographic characteristics, associations between non-employment and CMD component symptoms were calculated, stratified by age groups; these were adjusted for survey year. gender, financial crisis, marital status, tenure, socio-economic status, seen a doctor in the last 12 months, smoking status, at least at least one activities of daily living problems present and number of children. Furthermore, we additionally obtained population attributional fractions (PAFs) for each association – that is, the hypothetical percentage of cases (with each CMD component symptom) in the total population that might be prevented by removing the exposure to non-employment, thus taking into consideration both the strength of association and the prevalence of the exposure. Additional logistic regression models described adjusted associations of interest for all ages (16+ years) and for 25- to 54-year-olds as a core working age group, excluding those in full-time education or retired. Finally, fourteen CMD subcomponents were meta-analysed by each age group. In order to do this, initially meta-analysis was carried out using ln (OR) for each symptom and final meta-analysed ln (OR) was converted back to OR. Heterogeneity between symptoms for each age group were tested using I² statistic. The I² statistic describes the percentage of variation across studies that is due to heterogeneity rather than chance (Higgins et al., 2003).

## Results

Table 1 summarises the prevalence of CMD subcomponents by sociodemographic and socioeconomic factors. Highest prevalences were observed for sleep disturbance (29.9%), fatigue (28.7%), worry (19.4%) and irritability (18.2%). of the prevalence of somatic symptoms, fatigue, forgetfulness, worry about physical health, depression, anxiety and panic symptoms were highest in the 45- to 54-year age group, while sleep disturbance prevalence was highest in those aged 75 and over. The 16- to 24-year age group reported the highest prevalence of depressive ideas, irritability, worry, phobia, compulsive and obsessive symptoms. The prevalence of CMD subcomponents were all higher in the non-employed group, except for irritability which was higher in the paid employment group. Percentage prevalence common mental disorder component symptoms in each survey year by age is displayed in the Supplementary Table 1. In summary, highest prevalence of common mental disorder symptom in all age groups and all survey years generally was fatigue and sleep disturbance.

**Table 1.** Prevalences of common mental disorder component symptoms by socioeconomic characteristics among age 25 to 64 survey respondents.

Characteristics	Somatic	Fatigue	Forgetful	Sleep disturbance	Irritability	Worry about physical health	Depression	Depressive symptoms	Worry	Anxiety	Phobias	Panic	Compulsions	Obsessions
Number with symptoms	1,663	6,303	2,243	6,318	4,250	1,504	2,463	2,198	4,456	2,201	1,243	596	1,065	1,545
% with symptoms	7.8	29.7	10.6	29.7	20	7.1	11.6	10.3	21	10.4	5.8	2.8	5	7.3
Survey year														
1993	8.1	27.7	8.6	25.6	20.1	5	9.4	9	19.9	10	5	2.4	5.9	9.4
2000	8.2	30.1	11.4	30.7	21.5	7.6	12.4	10.8	20.9	10.4	5.4	2.5	3.5	6.2
2007	7.0	30.4	11.3	32.1	19.8	7.6	12.9	11.1	21.3	9.9	6.4	3.4	4.3	6.1
2014	7.8	32.2	12.5	33.7	17.8	9.9	13.2	11.5	22.8	11.6	7.7	3.3	6.3	5.9
Age														
25–34	7.2	31	10.2	26.9	25.2	5.5	11.4	11.1	22.5	10.1	6.6	2.6	6.3	8
35–44	8.3	29.3	10.9	28.1	22.9	6.4	11.8	11.2	22.1	10.4	6.6	3	5	7.9
45–54	9.1	31	12.2	32	18.5	8.4	12.7	11.3	22.1	11.8	5.6	3.2	4.6	7
55–64	6.6	27.2	8.9	32.2	12.8	8.1	10.5	7.6	17	9	4.4	2.4	4.1	6
Employment status														
In paid employment	6.3	26.5	8.1	25.7	18.3	4.9	8.7	7.7	18.9	8.2	4.2	1.7	4	5.8
Not in paid employment	11.5	37.4	16.6	39.6	24.2	12.3	18.7	16.9	26.1	15.6	9.8	5.5	7.4	10.8
Gender														
Female	9.4	34.3	11.9	34.5	22.2	7.3	12.3	12.1	23.3	11.4	7.3	3.3	5.7	8.6
Male	5.9	23.8	8.9	23.8	17.2	6.9	10.7	8.1	18	9	4	2.1	4.1	5.6
Financial crisis in the last 12 months	16	49.1	25.3	46.7	39.1	14.5	27.1	25.7	45.2	29.1	10.7	8.4	7.8	17.4
Marital status														
Divorced/separated/widowed	11.2	36.8	15.9	38.2	20.8	10.4	17.3	16.5	26.5	15.5	8.7	4.6	6.7	10.7
Married/cohabiting	6.8	27.1	8.3	26.8	19.6	5.7	9	7.7	18.5	8.4	4.2	2	3.9	6
Single	7.9	30.8	12.5	30.8	20.5	8.1	14.3	12.6	23.2	11.6	8.1	3.5	6.7	7.9
Tenure														
Owned outright	5.1	24.7	7.2	29.2	13	6	8.6	6.4	15.9	7.3	4	1.7	3.6	5.4
Owned with a mortgage	7.2	27.2	8.9	26.2	19.6	5.1	9.1	8.2	19.3	8.7	4.4	1.8	4	6.4
Private or other renter	10.6	37.6	16.8	37.7	25.7	12.1	18.2	17.5	28.2	15.3	9.9	5.2	7.6	9.5
Social housing	11.3	38.5	15.5	35.1	26	11	18.9	17.1	27.4	16.1	10	5.7	8.3	11.3
Socio-economic status														
Professional/managerial/clerical	7.4	28.5	9.5	28.4	19.1	5.7	9.8	9	20.5	9.4	4.9	2	4	6.6
Skilled/ partly-skilled occupations	7.4	29.6	10.5	29.7	20.7	7.6	12.6	11.2	20.1	10.5	6	3.2	5.9	7.8
Unskilled	12.4	39	18.7	40.5	23.2	14.8	20.8	17.2	28.2	16.3	11.8	7	9.1	9.6
Unknown	20	46.7	33.3	40	40	20	6.7	6.7	40	20	20	13.3	13.3	6.7
Seen a doctor in the last 12 months	9.9	35.6	13.1	34.3	23	9.5	13.5	12.4	24.1	12.4	6.9	3.6	5.6	8.5
Smoking status														
Current smoker	10.3	37.1	15.1	35.9	26	10.1	18	16.1	27.4	15.5	9.2	5	6.8	11
Ex-smoker	6.9	27.6	9.1	28.1	18.9	6	8.9	8.1	18.7	8.5	4.7	2	4.3	6.1
No smoker	7	26.2	8.5	27	16.3	5.9	9.6	8.3	18.5	8.4	4.5	2	4.4	5.6
Highest educational qualification														
A/Level and above	7.2	28.6	9.5	27.9	19.2	5.9	9.7	8.7	19.9	9.7	5	2.2	4.1	6.2
GCSE/GCE/O-level/other	7.4	29.4	10.6	29.2	20.7	6.7	11.2	10.2	21.1	9.7	6.2	2.6	5.1	7.4
No qualifications	9.6	32.1	12.6	34.2	20.5	10.3	16.1	13.9	22.8	12.7	7	4.4	6.8	9.3
At least 1 ADL present	15.7	53	24.7	48.4	30.8	18.1	25.9	22.7	33.9	21.5	13.4	8	9.2	12.7
Number of children														
None	7.7	28.9	10.2	30.8	16.5	7.7	11.8	9.9	20.4	10.6	5.8	2.9	5	7.1
1	8.5	33.1	11.7	29.7	24.4	6.3	11.7	12	21.9	10.1	6	2.7	5	8.2
2	7.6	28.6	10.8	25.6	27.1	5.5	10.3	9.8	21.4	9.1	5.3	2.3	5.1	7.1
3+	7.7	32.6	11.2	28.7	29.1	5.8	12.3	12.2	24.1	11.9	7.4	2.9	5	7.4

Considering other characteristics, female participants, current smokers and those who were private renters and living in social housing had higher prevalence of all CMD components than their respective counterparts. Nearly half of the respondents who reported a financial crisis in the last 12 months had fatigue (48.2%), sleep disturbance (45.9%) and worry (44.8%). In terms of marital status, those who were divorced or separated reported higher prevalence in most of the CMD subcomponents except irritability and compulsions, which were highest among those who were single, and sleep disturbance which was highest among those who were widowed. For socio-economic status, unskilled workers had highest prevalence of all CMD components except irritability and compulsions; those with no educational qualifications had highest prevalence of all components except irritability, worry and phobia. Those with five or more children had a higher prevalence of CMD components except anxiety and phobia. Finally, respondents who had seen a doctor in the last 12 months had the highest prevalence of fatigue and sleep symptoms, as did nearly half of the respondents who reported problems with at least one ADL impairment.

Table 2 summarises the strengths of associations between non-employment and CMD components by age group, accompanied by PAFs. Apart from the relatively rare obsessive and compulsive symptoms, odds ratios for associations with all CMD components were strongest in the 45 to 54 years age group compared to other age groups. However, for most components, the PAFs were higher in the 55 to 64 years age group, owing to higher prevalence of non-employment as an exposure in this group. Within the highest-risk 45 to 54 years age group, all odds ratios were statistically significant and strongest associations were observed with panic symptoms (OR = 2.33), followed by depressive symptoms (1.90), worry about physical health (1.84), depression (1.82), forgetfulness (1.82) and somatic symptoms (1.70). In the 55 to 64 years age group, highest population attributable fractions were observed for non-employment as a hypothetical risk factor for panic symptoms (51.7%), phobias (44.2%), forgetfulness (39.5%), depressive symptoms (38.5%), worries about physical health (37.9%) and somatic symptoms (36.0%).

**Table 2.** Fully adjusted^a^ associations between non-employment and CMD component symptoms, stratified by age group; odds ratios [95% CI] are displayed with population attributable fractions (%) for each outcome.

CMD component outcome	Age 25–34	Age 35–44	Age 45–54	Age 55–64
Somatic	1.21 [0.94, 1.57], 13.1	1.24 [0.97, 1.57], 16.4	1.70 [1.34, 2.15], 23.8	1.56 [1.20, 2.04], 36.0
Fatigue	1.04 [0.89, 1.21], 7.3	1.21 [1.03, 1.42], 10.3	1.23 [1.04, 1.45], 12.9	0.99 [0.86, 1.15], 17.8
Forgetful	1.28 [1.02, 1.61], 16.9	1.45 [1.17, 1.79], 20.6	1.82 [1.47, 2.25], 28.5	1.56 [1.18, 1.90], 39.5
Sleep	1.29 [1.10, 1.51], 10.6	1.19 [1.02, 1.40], 9.7	1.63 [1.39, 1.91], 14.1	1.30 [1.13, 1.49], 19.6
Irritability	1.37 [1.17, 1.61], 11.8	1.16 [0.98, 1.37], 7.8	1.38 [1.15, 1.66], 13.9	1.10 [0.91, 1.33], 16.9
Worry about physical health	1.22 [0.91, 1.63], 16.5	1.55 [1.19, 2.02], 25.5	1.84 [1.44, 2.36], 36.0	1.38 [1.08, 1.76], 37.9
Depression	1.36 [1.10, 1.68], 19.4	1.75 [1.42, 2.14], 24.2	1.82 [1.48, 2.24], 31.4	1.24 [1.00, 1.54], 28.5
Depressive symptoms	1.58 [1.28, 1.96], 22.7	1.63 [1.32, 2.00], 25.2	1.90 [1.53, 2.35], 30.3	1.47 [1.14, 1.90], 38.5
Worry	1.10 [0.93, 1.31], 8.4	1.25 [1.06, 1.48], 10.4	1.37 [1.15, 1.63], 14.1	1.04 [0.88, 1.24], 16.1
Anxiety	1.51 [1.21, 1.89], 17.8	1.60 [1.29, 1.99], 21.0	1.61 [1.30, 2.00], 24.1	1.12 [0.90, 1.41], 25.2
Phobias	1.70 [1.31, 2.21], 25.9	1.34 [1.04, 1.74], 23.1	1.62 [1.20, 2.19], 34.7	1.50 [1.08, 2.08], 44.2
Panic	1.64 [1.09, 2.45], 33.9	1.44 [1.00, 2.07], 33.6	2.33 [1.58, 3.43], 48.9	1.63 [1.02, 2.61], 51.7
Compulsions	1.43 [1.09, 1.88], 19.2	1.30 [0.97, 1.74], 19.2	1.39 [1.00, 1.92], 24.5	0.95 [0.69, 1.3], 20.0
Obsessions	1.35 [1.06, 1.73], 15.0	1.85 [1.47, 2.34], 23.7	1.65 [1.27, 2.15], 25.1	1.24 [0.95, 1.62], 23.1

a Adjusted for survey year, gender, financial crisis, marital status, tenure, socio-economic status, seen a doctor in the last 12 months, smoking status, at least at least one activities of daily living problems present and number of children.

Associations between non-employment and CMD component symptoms in all age groups, and in the 25 to 54 years peak working age range, are further described in Table 3. In the second of these, odds ratios are ranked similarly in strength to those previously described for the 45 to 54 years age group, although associations with anxiety and obsessive symptoms emerge as relatively strong, owing to associations in younger age groups. PAFs are ranked similarly to those in the 55 to 64 years age range described above, although those for somatic symptoms are less important across the broader age range.

**Table 3.** Fully adjusted^a^ odds ratios [95% CI] and population attributable fractions (PAF) for associations between non-employment and CMD component symptoms among age 25 to 54 years age group.

CMD component symptom	OR [95% CI], PAF
Somatic	1.34 [1.17, 1.55], 18.0
Fatigue	1.15 [1.04, 1.26], 10.2
Forgetful	1.46 [1.29, 1.65], 22.2
Sleep	1.32 [1.20, 1.44], 11.5
Irritability	1.29 [1.17, 1.42], 10.9
Worry about physical health	1.52 [1.31, 1.77], 27.2
Depression	1.61 [1.43, 1.81], 25.1
Depressive symptoms	1.65 [1.46, 1.86], 26.1
Worry	1.20 [1.09, 1.32], 10.9
Anxiety	1.50 [1.33, 1.7], 21.1
Phobias	1.51 [1.30, 1.77], 27.3
Panic	1.71 [1.38, 2.13], 39.0
Compulsions	1.37 [1.16, 1.62], 20.7
Obsessions	1.57 [1.37, 1.81], 21.2

a Adjusted for survey year, gender, financial crisis, marital status, tenure, socio-economic status, seen a doctor in the last 12 months, smoking status, at least at least one activities of daily living problems present and number of children.

In the meta-analysis investigating heterogeneity by outcome (Table 4), associations with non-employed status were found to have high heterogeneity (I^2^: 81.6%) across the 14 component symptoms both in the total sample and in the 25 to 54 years working age range. Considering the more specific age strata, this heterogeneity was highest in the 35 to 44, 45 to 54 and 55 to 64 years groups compared to those in younger or older age groups.

**Table 4.** Meta-analysis output for age-group-specific associations between non-employment and the 14 CMD component symptoms.

Age group	Meta analysed (OR; Lower CI, Upper CI)	*I*^2^ (%)	*p* value for heterogeneity^a^
All age	1.30 [1.22, 1.39]	81.6	<.001
25–54	1.45 [1.32, 1.57]	83.7	<.001
25–34	1.32 [1.22, 1.57]	45.1	.03
35–44	1.40 [1.28, 1.52]	54.9	.01
45–54	1.61 [1.48, 1.75]	50.6	.02
55–64	1.24 [1.13, 1.36]	56.0	.01

a *I*^2^ heterogeneity tests applied to the log ORs.

In the meta-analysis investigating heterogeneity by age groups (Supplemental Table 2), highest heterogeneity levels were found for sleep problems, worry about physical health, depression, phobias and somatic symptoms. *I*^2^ coefficients were reduced but still showing the same pattern when the sample was restricted to the 25 to 54 years age range. Outcome-specific heterogeneity by age group is further displayed in Supplemental Figure 1.

## Discussion

Our study investigated the associations between non-employed status and a wide range of CMD component symptoms, evaluating both the level of heterogeneity in associations with different symptoms and that across age groups. Associations measured by odds ratios were strongest in the 45 to 54 years age group, while PAFs were generally highest in the 55 to 64 years age group. Variation of associations with the different outcomes was highest in the middle of the age groups compared to younger or older groups and there was also higher age group heterogeneity for associations with non-employment in some CMD symptoms than others.

Our study found a higher prevalence of all CMD components in non-employed people in the working age range of 25 to 54 years. This is consistent with previously reported findings from England in a sample aged 21 to 54 years reporting a higher prevalence of CMD overall among those who were not employed (Butterworth et al., 2012), as well as in a study in Catalonia, Spain, among respondents aged 25 to 64 years (Artazcoz et al., 2004) and a study in Bosnia and Herzegovina among respondents aged 23 to 65 years (Batic-Mujanovic et al., 2017). CMD, however, is quite a broad entity, encompassing a range of symptoms of anxiety and depression. On the one hand, the impact of non-employment may be only on a single underlying CMD construct resulting in similar associations with all component symptoms; on the other hand, previously reported associations with CMD might be accounted for by specific associations with particular symptoms. Our findings support a position in between, in that associations were present with all component symptoms but stronger for some than others. In this respect, data from the 1992 British Household Panel Study (BHPS) found unemployed status to be associated with symptoms suggestive of depression including inability to enjoy day-to-day activities, feeling of worthlessness and less happiness, among age groups 25 to 65. (Theodossiou, 1998). Other symptoms associated with non-employment have included depression and depressive symptoms (Bedaso et al., 2021; Caetano et al., 2016; Honkonen et al., 2007; Theodossiou, 1998), memory and cognition (Xue et al., 2017), insomnia (Palmer et al., 2017; Wolińska, 2020) and panic disorder (Honkonen et al., 2007). Our findings in the highest risk (45–54 years) age group of particularly strong associations with panic and depressive symptoms are consistent with this. Associations with somatic symptoms and worries about physical health may reflect co-existing health conditions associated with non-employment in this group, and subjective cognitive symptoms might reflect the loss of routine because of non-employment. The relative lack of associations with fatigue across the age groups may reflect higher levels of fatigue associated with CMD in people who are in more demanding employment and that might be mitigated by non-employment. However, these patterns require confirmation in independent samples.

Several processes may underlie the observed contemporaneous associations between non-employment and CMD symptoms. First, preceding mental health problems may be causes of non-employment (Dooley et al., 2000; Owen et al., 1995). Second, common risk factors may contribute to both non-employment and CMD (Daly et al., 2013); for example, factors like low education and substance misuse may increase both the risk of mental health problems (Friedman et al., 2009) as well as loss of employment or difficulties attaining it (Clark & Lepinteur, 2019). However, evidence from previous literature suggests a causal link from non-employment towards CMD in that studies have frequently found that non-employed status precedes mental health problems (Zhang et al., 2013) and is associated with a higher prevalence of poor mental health following other stressors (Bosmans et al., 2018), as well as an increase in antidepressant purchases following retirement (Olesen et al., 2015). Non-employment is associated with an increased risk of several health-threatening lifestyles including smoking (Janlert, 1997; Jung et al., 2013), higher alcohol consumption (Bedaso et al., 2021; Honkonen et al., 2007) and weight gain (Morris et al., 1992). Most of these were also risk factors for sleep difficulties (Benbir et al., 2015; Pearson et al., 2006) and depression (Dong et al., 2004; Salokangas et al., 1998). Physical activity, known to be associated with better mental health (Biddle, 2016), may also be implicated. Other factors mediating the risk of CMD in non-employed groups naturally include economic hardships (Acarturk et al., 2020).

Our study specifically investigated variation by age group in associations of non-employment with CMD or its symptoms. I^2^ analysis revealed significant heterogeneity across age groups for several symptoms: somatic, sleep, worries about physical health, depression and phobias. These were primarily explained by stronger associations in mid-life with weak or absent associations in older age groups and, sometimes in younger age groups. The different meaning of non-employment before and after retirement is an obvious explanation for heterogeneity, and statistics were diminished in strength and significance when the sample was restricted to those aged 25 to 54 years, although the coefficient for sleep difficulties remained significant. Considering other investigations of age-specificity of associations, a similar study of respondents aged 18 to 55 years in Canada, found an association between unemployment and distress only in those aged 31 to 55 years (Breslin et al., 2003). A study of participants aged 19 to 63 years found poorest mental health among those unemployed in age group 35 to 44 years and significantly better subjective well-being in the 55 to 65 years age group (Hepworth, 1980). Mental health outcomes therefore appear to be worse for those unemployed in middle-age compared to younger or older adults. There are several potential reasons for this. First, a lack of pre-unemployment worktime exposure and shorter-duration periods of unemployment might explain weaker associations in younger adults. In this respect worse mental health outcomes have been found in unemployed people with work experiences of more than 5 years compared to those with no work experience (Batic-Mujanovic et al., 2017). In addition, an English study found a significant risk of CMD in those less than one year or greater than three years post-unemployment (Ford et al., 2010). Second, there will be variation by age in causes of non-employment. In this respect, involuntary retirement is associated with a significantly higher risk of poor mental health (Batic-Mujanovic et al., 2017; Dave et al., 2006) and may be more common in the 45 to 54 years age group where associations were strongest. Bearing in mind the cross-sectional findings, CMD risk factors like personality problems or a physical disability might also exert particular effects in this age group compared to younger and older groups. Finally, the pressures arising from non-employment would be expected to be higher in middle-aged groups because of additional responsibilities, and economic hardship is a well-recognised risk factor for CMD (Acarturk et al., 2020). The strength of association between non-employment and sleep symptoms in unemployed varied strongly between age groups, even when the youngest and oldest groups were excluded, due to a particularly strong association in the 45 to 54 years age group. This might again reflect the reason for non-employment here (e.g. underlying physical health problems resulting in early retirement) or the impact of non-employment in an age group likely to have other financial responsibilities. Also, older and younger age groups might have other competing causes of sleep disturbance (some possibly employment-related) obscuring the association. Of relevance to post-retirement associations, previous research has found that older age groups receive more benefits from retirement on sleep problems than younger age groups (Marquiáe et al., 2012).

Our study had several strengths including its large sample size of over 28,000 respondents drawn to be nationally representative and covering a number of survey years. To our knowledge our study is the only study to compare associations of non-employment with different components of CMD, to investigate differences between age groups, and to include formal assessment of heterogeneity. Furthermore, we believe the PAF estimations to be unique. These, however, need to be viewed with caution as they presuppose unconfounded associations and unidirectional causal relationships, which clearly cannot be assumed from cross-sectional analyses; therefore, they remain hypothetical, although a helpful means of comparing potential impact in addition to strength of association. The cross-sectional associations further preclude any firm conclusions on the direction of causality. Additional considerations include the multiple analyses because of different outcomes and age stratification, although conclusions have been drawn on what appeared to be consistency in patterns of associations rather than on individual estimates. Finally, potential confounding factors were limited to those on which data were available across the multiple survey waves pooled for this analysis, and we did not attempt further analyses of underlying reasons for non-employed status which may clearly vary between individuals.

We have sought to provide a comprehensive picture on variations in the level of importance of non-employment vs. employment on different mental health symptoms in different age groups. The particularly high impact in middle-aged, pre-retirement groups suggests a policy focus on alleviating stressors and providing support for those made redundant and/or compelled to take unwanted early retirement.

## Supplemental Material

sj-docx-1-isp-10.1177_00207640241293351 – Supplemental material for Associations of non-employment with common mental disorder subcomponents among working age population: analysis of national data from 1993, 2000, 2007 and 2014 Supplemental material, sj-docx-1-isp-10.1177_00207640241293351 for Associations of non-employment with common mental disorder subcomponents among working age population: analysis of national data from 1993, 2000, 2007 and 2014 by Gayan Perera, KTDDP Jayapala, Mizanur Khondoker, Karen Glaser, Giorgio Di Gessa and Robert Stewart in International Journal of Social Psychiatry
