# Supplementary material for: Associations of non-employment with common mental disorder subcomponents among working age population: Analysis of national data from 1993, 2000, 2007 and 2014
Source: Int J Soc Psychiatry. 2024 Nov 14;71(2):404–13. doi: 10.1177/00207640241293351 (PMC11874606; doi:10.1177/00207640241293351)
Supplement: sj-docx-1-isp-10.1177_00207640241293351 – Supplemental material for Associations of non-employment with common mental disorder subcomponents among working age population: analysis of national data from 1993, 2000, 2007 and 2014 [file sj-docx-1-isp-10.1177_00207640241293351.docx]

**Supplementary table 1:** **Percentage prevalence common mental disorder component symptoms in each survey year by age**

| Survey year | Age | Somatic | Fatigue | Forgetful | Sleep disturbance | Irritability | Worry about physical health | Depression | Depressive symptoms | Worry | Anxiety | Phobias | Panic | Compulsions | Obsessions |
| --- | --- | --- | --- | --- | --- | --- | --- | --- | --- | --- | --- | --- | --- | --- | --- |
| Survey year |  |  |  |  |  |  |  |  |  |  |  |  |  |  |  |
| 1993 | 25- 34 | 7.0 | 29.9 | 8.5 | 23.0 | 26.9 | 4.2 | 9.8 | 9.6 | 21.6 | 9.7 | 6.0 | 2.5 | 6.8 | 9.9 |
|  | 35- 44 | 8.7 | 26.9 | 8.6 | 24.5 | 22.4 | 5.2 | 9.8 | 9.9 | 22.4 | 9.7 | 5.5 | 2.9 | 5.7 | 10.5 |
|  | 45- 54 | 9.8 | 27.4 | 9.8 | 27.0 | 17.1 | 5.4 | 10.0 | 9.8 | 19.2 | 10.9 | 3.9 | 2.4 | 5.4 | 9.6 |
|  | 55- 64 | 7.2 | 25.8 | 7.5 | 29.0 | 11.6 | 5.4 | 7.9 | 6.3 | 15.5 | 9.9 | 4.2 | 1.7 | 5.5 | 7.3 |
|  |  |  |  |  |  |  |  |  |  |  |  |  |  |  |  |
| 2000 | 25- 34 | 7.5 | 31.3 | 11.2 | 28.9 | 26.1 | 6.5 | 12.2 | 11.3 | 22.7 | 9.8 | 6.0 | 1.9 | 4.3 | 7.2 |
|  | 35- 44 | 8.8 | 30.2 | 11.6 | 29.8 | 24.1 | 7.1 | 13.7 | 12.3 | 22.0 | 10.4 | 6.3 | 2.7 | 3.6 | 6.9 |
|  | 45- 54 | 9.4 | 31.8 | 12.8 | 32.3 | 20.0 | 8.3 | 12.3 | 10.6 | 21.8 | 12.6 | 5.5 | 2.9 | 3.1 | 5.2 |
|  | 55- 64 | 6.7 | 26.6 | 9.5 | 32.2 | 14.3 | 8.9 | 10.8 | 8.5 | 16.5 | 8.5 | 3.2 | 2.4 | 2.8 | 5.3 |
|  |  |  |  |  |  |  |  |  |  |  |  |  |  |  |  |
| 2007 | 25- 34 | 7.5 | 30.5 | 11.3 | 31.8 | 23.8 | 6.3 | 12.4 | 12.5 | 22.8 | 9.9 | 7.5 | 3.6 | 5.8 | 6.9 |
|  | 35- 44 | 6.3 | 30.0 | 11.8 | 28.9 | 23.3 | 6.5 | 11.6 | 11.0 | 21.4 | 10.3 | 7.3 | 3.4 | 5.0 | 6.4 |
|  | 45- 54 | 9.0 | 33.1 | 13.3 | 35.0 | 19.7 | 9.6 | 16.5 | 14.7 | 24.7 | 12.0 | 6.5 | 4.1 | 3.8 | 6.0 |
|  | 55- 64 | 5.6 | 28.5 | 8.8 | 33.3 | 12.7 | 8.0 | 11.5 | 6.9 | 17.0 | 7.5 | 4.5 | 2.7 | 2.9 | 5.2 |
|  |  |  |  |  |  |  |  |  |  |  |  |  |  |  |  |
| 2014 | 25- 34 | 7.1 | 34.3 | 11.8 | 28.4 | 19.9 | 6.8 | 12.9 | 13.1 | 24.5 | 12.5 | 8.6 | 2.7 | 9.1 | 5.0 |
|  | 35- 44 | 9.7 | 32.0 | 13.6 | 31.6 | 21.3 | 8.0 | 12.9 | 12.4 | 22.6 | 12.1 | 8.7 | 3.1 | 6.0 | 6.5 |
|  | 45- 54 | 7.6 | 33.9 | 13.9 | 36.9 | 17.6 | 12.1 | 13.5 | 11.3 | 24.3 | 12.1 | 7.5 | 4.1 | 5.8 | 6.2 |
|  | 55- 64 | 7.0 | 28.8 | 10.6 | 36.1 | 13.4 | 11.7 | 13.3 | 9.7 | 20.0 | 10.1 | 6.2 | 3.1 | 5.0 | 5.7 |

**Supplementary table 2: Meta-analyses of associations between non-employment and each CMD component symptom across the age groups of the sample**

|  | Age 25- 54 | | |
| --- | --- | --- | --- |
| Symptom | Meta analysed OR (Lower CI, Upper CI) | I^2^ | P value |
| Somatic | 1.35 (0.94, 1.54) | 44.1% | 0.15 |
| Fatigue | 1.15 (0.86, 1.25) | 0.0% | 0.45 |
| forgetful | 1.56 (0.99, 1.75) | 57.0% | 0.07 |
| Sleep | 1.39 (1.27, 1.51) | 68.7% | **0.02** |
| Irritability | 1.28 (0.87, 1.4) | 1.7% | 0.38 |
| Worry about physical health | 1.51 (0.92, 1.74) | 46.7% | 0.13 |
| Depression | 1.64 (0.90, 1.84) | 32.0% | 0.22 |
| Depressive symptoms | 1.73 (0.73, 1.94) | 0.0% | 0.49 |
| Worry | 1.24 (0.82, 1.36) | 6.7% | 0.36 |
| Anxiety | 1.56 (0.48, 1.76) | 0.0% | 0.96 |
| Phobias | 1.47 (0.84, 1.69) | 22.0% | 0.28 |
| Panic | 1.81 (0.85, 2.22) | 20.8% | 0.29 |
| Compulsions | 1.36 (0.53, 1.59) | 0.0% | 0.97 |
| Obsessions | 1.63 (0.76, 1.86) | 15.5% | 0.31 |

**Supplementary Figure 1: Associations between non-employment and CMD component symptoms by age group (25-64 years)**
